# Supplementary figures and images for: Treatment and control of blood pressure in Welsh patients with and without depression: A study of whole-population electronic health records
Source: PLoS One. 2025 Jun 25;20(6):e0326583. doi: 10.1371/journal.pone.0326583 (PMC12192142; doi:10.1371/journal.pone.0326583)

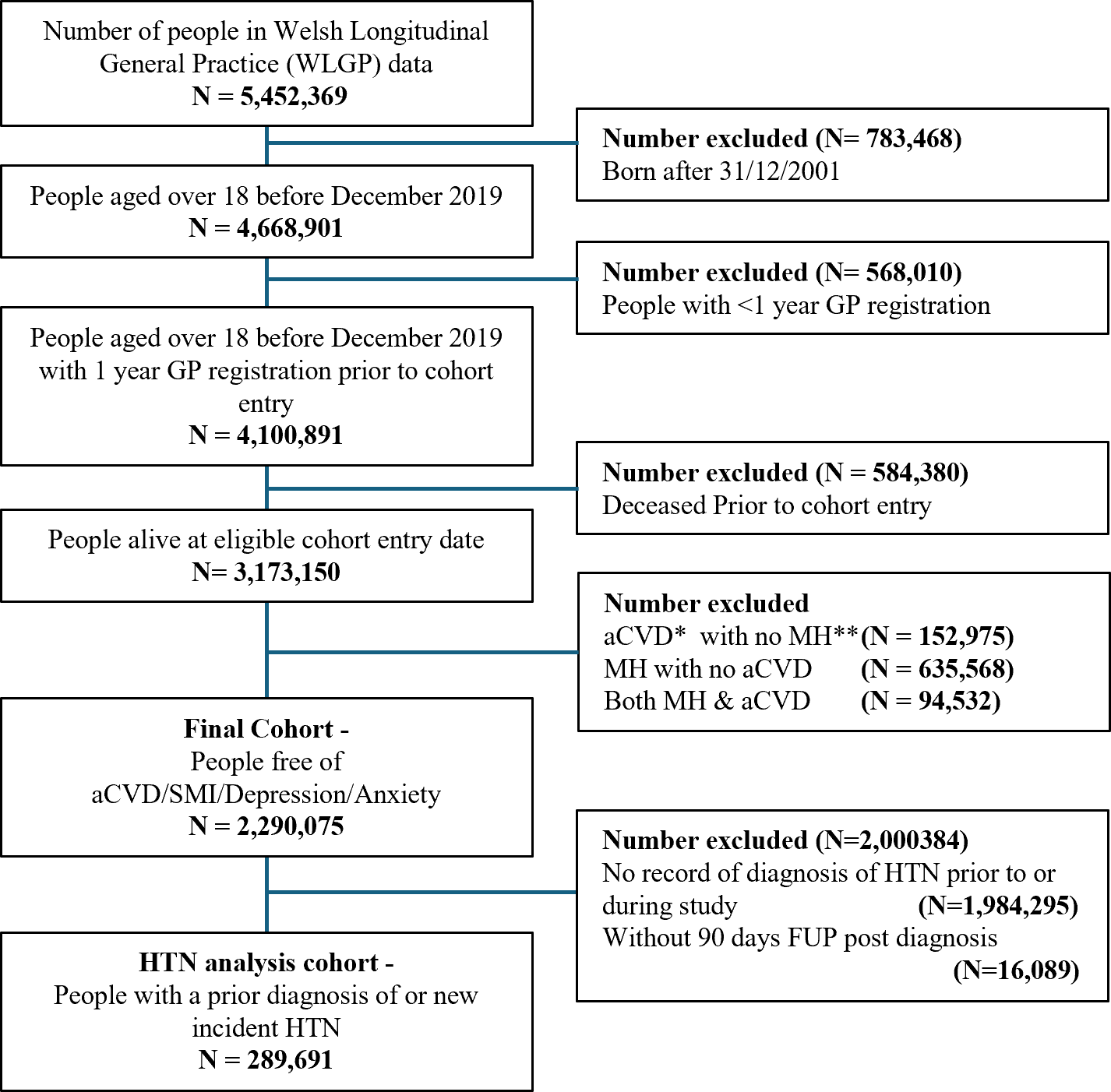

Supplement: S1 Fig — (TIF) [file pone.0326583.s001.tif]
