# Supplementary material for: Treatment and control of blood pressure in Welsh patients with and without depression: A study of whole-population electronic health records
Source: PLoS One. 2025 Jun 25;20(6):e0326583. doi: 10.1371/journal.pone.0326583 (PMC12192142; doi:10.1371/journal.pone.0326583)
Supplement: S1 Table — (DOCX) [file pone.0326583.s002.docx]

**Supplement Table 1.** Characteristics of patients with a prior diagnosis of hypertension with and without a valid follow-up blood pressure assessment

|  | **Valid blood pressure** | **No blood pressure** | **p** |
| --- | --- | --- | --- |
| n population | 181174 (76.3) | 56261 (23.7) |  |
| Depressed y (n%) | 15406 (8.5) | 3709 (6.6) | <0.001 |
| mean age y (SD) | 65.3 (12.9) | 64.0 (15.0) | <0.001 |
| Characteristic n (%) |  |  |  |
| Female | 91330 (50.4) | 29200 (51.9) | <0.001 |
| Deprivation index (233822) |  |  | <0.001 |
| 1 (most deprived) | 31440 (17.6) | 9385 (17.0) |  |
| 2 | 35152 (19.7) | 10974 (19.9) |  |
| 3 | 39319 (22.0) | 11258 (20.4) |  |
| 4 | 35678 (20.0) | 12739 (23.1) |  |
| 5 (least deprived) | 37034 (20.7) | 10843 (19.6) |  |
|  |  |  |  |
| Geographic location (235126) |  |  | <0.001 |
| Rural | 60337 (33.6) | 20707 (37.2) |  |
| Urban | 119184 (66.4) | 34898 (62.8) |  |
|  |  |  |  |
| Dyslipidaemia | 36351 (20.1) | 7974 (14.2) | <0.001 |
| Diabetes Mellitus | 33273 (18.4) | 7162 (12.7) | <0.001 |
| CKD | 2133 (1.2) | 620 (1.1) | 0.15 |
| Liver disease | 2300 (1.3) | 807 (1.4) | 0.003 |
| Cancer | 22264 (12.3) | 6462 (11.5) | <0.001 |
| Antihypertensive therapy | 163430 (90.2) | 17378 (30.9) | <0.001 |
