# Supplementary material for: Treatment and control of blood pressure in Welsh patients with and without depression: A study of whole-population electronic health records
Source: PLoS One. 2025 Jun 25;20(6):e0326583. doi: 10.1371/journal.pone.0326583 (PMC12192142; doi:10.1371/journal.pone.0326583)
Supplement: S2 Table — (DOCX) [file pone.0326583.s003.docx]

**Supplement Table 2.** Unadjusted binary logistic regression estimates of predictors of A) being prescribed antihypertensive therapy and B) blood pressure control in patients with a prior diagnosis of hypertension

| **A** | **OR** | **95% C.I.** | **p** |  | **B** | **OR** | **95% C.I.** | **p** |  |
| --- | --- | --- | --- | --- | --- | --- | --- | --- | --- |
| Depression | 1.55 | 1.49 - 1.61 | <0.001 |  | Depression | 1.47 | 1.42 - 1.52 | <0.001 | |
| Age group |  |  | <0.001 |  | Age group |  |  | <0.001 | |
| 40-59 | 2.95 | 2.82 - 3.08 | <0.001 |  | 40-59 | 0.84 | 0.79 - 0.88 | <0.001 | |
| 60-74 | 3.63 | 3.48 - 3.80 | <0.001 |  | 60-74 | 0.77 | 0.73 - 0.81 | <0.001 | |
| 75+ | 2.82 | 2.70 - 2.95 | <0.001 |  | 75+ | 0.71 | 0.67 - 0.75 | <0.001 | |
| Female | 0.87 | 0.85 - 0.88 | <0.001 |  | Female | 1.06 | 1.04 - 1.08 | <0.001 | |
| Deprivation quintiles WIMD |  |  | <0.001 |  | Deprivation quintiles WIMD |  |  | <0.001 | |
| 2 | 0.86 | 0.83 - 0.89 | <0.001 |  | 2 | 0.96 | 0.94 - 0.99 | 0.020 | |
| 3 | 0.94 | 0.91 -0.97 | <0.001 |  | 3 | 0.94 | 0.91 - 0.97 | <0.001 | |
| 4 | 0.72 | 0.70 - 0.75 | <0.001 |  | 4 | 0.95 | 0.92 - 0.98 | <0.001 | |
| 5 (least deprived) | 0.91 | 0.89 - 0.94 | <0.001 |  | 5 (least deprived) | 0.96 | 0.94 - 0.99 | 0.015 | |
| Location of residence: Urban | 1.15 | 1.12 - 1.17 | <0.001 |  | Location of residence: Urban | 0.95 | 0.93 - 0.97 | <0.001 | |
| History of diabetes | 1.46 | 1.42- 1.50 | <0.001 |  | History of diabetes | 1.34 | 1.31 - 1.37 | <0.001 | |
| History of chronic kidney disease | 1.06 | 0.97 - 1.16 | 0.22 |  | History of chronic kidney disease | 1.43 | 1.31 - 1.56 | <0.001 | |
| History of dyslipidaemia | 1.56 | 1.52 - -1.60 | <0.001 |  | History of dyslipidaemia | 1.06 | 1.04 - 1.09 | <0.001 | |
| History of liver disease | 0.80 | 0.74 - 0.87 | <0.001 |  | History of liver disease | 1.16 | 1.07 - 1.26 | <0.001 | |
| History of cancer | 1.01 | 0.98 - 1.04 | 0.40 |  | History of cancer | 1.04 | 1.01 - 1.07 | 0.014 | |
|  |  |  |  |  | Antihypertensive therapy within 1 year | 1.05 | 1.02 - 1.08 | 0.003 | |
