# Supplementary material for: Treatment and control of blood pressure in Welsh patients with and without depression: A study of whole-population electronic health records
Source: PLoS One. 2025 Jun 25;20(6):e0326583. doi: 10.1371/journal.pone.0326583 (PMC12192142; doi:10.1371/journal.pone.0326583)
Supplement: S3 Table — (DOCX) [file pone.0326583.s004.docx]

**Supplement Table 3.** Characteristics of patients with new incident hypertension with and without a valid follow-up blood pressure assessment

|  | **Valid blood pressure** | **No blood pressure** | **p** |
| --- | --- | --- | --- |
| n population | 19327 (37.0) | 32929 (63.0) |  |
| Depressed y (n%) | 1042 (5.4) | 360 (1.1) | <0.001 |
| mean age y (SD) | 58.2 (14.2) | 65.7 (14.1) | <0.001 |
| Characteristic n (%) |  |  |  |
| Female | 8682 (44.9) | 16746 (50.9) | <0.001 |
| Deprivation index (51254) |  |  | <0.001 |
| 1 (most deprived) | 3532 (18.5) | 4220 (13.1) |  |
| 2 | 3954 (20.8) | 6112 (19.0) |  |
| 3 | 4078 (21.4) | 6102 (18.9) |  |
| 4 | 3650 (19.2) | 9241 (28.7) |  |
| 5 (least deprived) | 3828 (20.1) | 6537 (20.3) |  |
|  |  |  |  |
| Geographic location (51656) |  |  | <0.001 |
| Rural | 6104 (31.8) | 14987 (46.2) |  |
| Urban | 13080 (68.2) | 17485 (53.8) |  |
|  |  |  |  |
| Dyslipidaemia | 1549 (8.0) | 1171 (3.6) | <0.001 |
| Diabetes Mellitus | 2083 (10.8) | 2015 (6.1) | <0.001 |
| Chronic Kidney Disease | 95 (0.5) | 166 (0.5) | 0.84 |
| Liver disease | 160 (0.8) | 243 (0.7) | 0.26 |
| Cancer | 1866 (9.7) | 3221 (9.8) | 0.64 |
| Antihypertensive therapy | 15085 (78.1) | 1873 (5.7) | <0.001 |
