# Supplementary material for: Treatment and control of blood pressure in Welsh patients with and without depression: A study of whole-population electronic health records
Source: PLoS One. 2025 Jun 25;20(6):e0326583. doi: 10.1371/journal.pone.0326583 (PMC12192142; doi:10.1371/journal.pone.0326583)
Supplement: S4 Table — (DOCX) [file pone.0326583.s005.docx]

**Supplement Table 4.** Unadjusted binary logistic estimates of predictors of A) being prescribed antihypertensive therapy and B) blood pressure control in patients with new incident hypertension

| **A** | **OR** | **95% C.I.** | **p** |  | **B** | **OR** | **95% C.I.** | **p** |
| --- | --- | --- | --- | --- | --- | --- | --- | --- |
| Depression | 3.82 | 3.42-4.27 | <0.001 |  | Depression | 1.31 | 1.15 -1.49 | <0.001 |
| Age group |  |  | <0.001 |  | Age group |  |  | <0.001 |
| 40-59 | 1.03 | 0.96-1.11 | 0.38 |  | 40-59 | 0.75 | 0.68 - 0.83 | <0.001 |
| 60-74 | 0.53 | 0.49-0.57 | <0.001 |  | 60-74 | 0.81 | 0.73 - 0.90 | <0.001 |
| 75+ | 0.29 | 0.27-0.31 | <0.001 |  | 75+ | 0.97 | 0.86 - 1.10 | 0.64 |
| Female | 0.72 | 0.70-0.75 | <0.001 |  | Female | 1.26 | 1.19 - 1.33 | <0.001 |
| Deprivation quintiles WIMD |  |  | <0.001 |  | Deprivation quintiles WIMD |  |  | 0.002 |
| 2 | 0.70 | 0.66-0.75 | <0.001 |  | 2 | 1.08 | 0.98 - 1.18 | 0.11 |
| 3 | 0.74 | 0.69-0.78 | <0.001 |  | 3 | 1.07 | 0.98 - 1.17 | 0.14 |
| 4 | 0.46 | 0.43-0.48 | <0.001 |  | 4 | 1.15 | 1.04 - 1.26 | 0.004 |
| 5 (least deprived) | 0.69 | 0.65-0.73 | <0.001 |  | 5 (least deprived) | 1.19 | 1.09 - 1.31 | <0.001 |
| Location of residence: Urban | 1.72 | 1.66-1.79 | <0.001 |  | Location of residence: Urban | 0.99 | 0.93 - 1.05 | 0.71 |
| History of diabetes | 1.56 | 1.46-1.66 | <0.001 |  | History of diabetes | 1.84 | 1.67 - 2.03 | <0.001 |
| History of chronic kidney disease | 1.06 | 0.82-1.37 | 0.66 |  | History of chronic kidney disease | 2.79 | 1.75 - 4.47 | <0.001 |
| History of dyslipidaemia | 2.30 | 2.13-2.48 | <0.001 |  | History of dyslipidaemia | 1.38 | 1.24 - 1.53 | <0.001 |
| History of liver disease | 1.26 | 1.03-1.55 | 0.024 |  | History of liver disease | 1.57 | 1.13 - 2.17 | 0.006 |
| History of cancer | 1.02 | 0.95-1.08 | 0.63 |  | History of cancer | 1.26 | 1.14 - 1.39 | <0.001 |
|  |  |  |  |  | Antihypertensive therapy within 1 year | 0.77 | 0.72 -0.83 | <0.001 |
